# Implications for the mesopelagic microbial gardening hypothesis as determined by experimental fragmentation of Antarctic krill fecal pellets

**DOI:** 10.1002/ece3.7119

**Published:** 2020-12-28

**Authors:** Emma L. Cavan, So Kawaguchi, Philip W. Boyd

**Affiliations:** ^1^ Institute for Marine and Antarctic Studies University of Tasmania Battery Point TAS Australia; ^2^ Department of Life Sciences, Silwood Park Campus Imperial College London Ascot UK; ^3^ Australian Antarctic Division Kingston TAS Australia; ^4^ Antarctic Climate and Ecosystems CRC University of Tasmania Battery Point TAS Australia

**Keywords:** carbon sink, fecal pellets, krill, mesopelagic zone, microbial gardening, zooplankton

## Abstract

1. Detritivores need to upgrade their food to increase its nutritional value. One method is to fragment detritus promoting the colonization of nutrient‐rich microbes, which consumers then ingest along with the detritus; so‐called microbial gardening. Observations and numerical models of the detritus‐dominated ocean mesopelagic zone have suggested microbial gardening by zooplankton is a fundamental process in the ocean carbon cycle leading to increased respiration of carbon‐rich detritus. However, no experimental evidence exists to demonstrate that microbial respiration rates are higher on recently fragmented sinking detrital particles.
2. Using aquaria‐reared Antarctic krill fecal pellets, we showed fragmentation increased microbial particulate organic carbon (POC) turnover by 1.9×, but only on brown fecal pellets, formed from the consumption of other pellets. Microbial POC turnover on un‐ and fragmented green fecal pellets, formed from consuming fresh phytoplankton, was equal. Thus, POC content, fragmentation, and potentially nutritional value together drive POC turnover rates.
3. Mesopelagic microbial gardening could be a risky strategy, as the dominant detrital food source is settling particles; even though fragmentation decreases particle size and sinking rate, it is unlikely that an organism would remain with the particle long enough to nutritionally benefit from attached microbes. We propose “communal gardening” occurs whereby additional mesopelagic organisms nearby or below the site of fragmentation consume the particle and the colonized microbes.
4. To determine how fragmentation impacts the remineralization of sinking carbon‐rich detritus and to parameterize microbial gardening in mesopelagic carbon models, three key metrics from further controlled experiments and observations are needed; how particle composition (here, pellet color/krill diet) impacts the response of microbes to the fragmentation of particles; the nutritional benefit to zooplankton from ingesting microbes after fragmentation along with identification of which essential nutrients are being targeted; how both these factors vary between physical (shear) and biological particle fragmentation.

## Graphical abstract

We present results from the first experiment on ocean mesopelagic zone microbial gardening. Marine detritivores would benefit from fragmenting only the most detrital particles as only these resulted in increases in microbial activity (and thus essential nutrients for detritivores). As microbial gardening is an important carbon feedback in the ocean, carbon cycling models need to parameterize microbial activity using not just the size of the particle, but also nutrient content, to simulate this process.

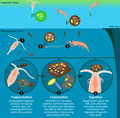

## 1 INTRODUCTION

Sinking detrital particulate organic carbon (POC) in the ocean is a vital sink for atmospheric carbon, without which there would be 50% more CO_2_ in the atmosphere than at present (Parekh et al., 2005; Volk & Hoffert, 1985). Carbon sinks to depth predominantly as phytodetrital aggregates made of dead/living phytoplankton cells, the fecal pellets of small pelagic crustaceans called zooplankton or zooplankton discards such as *Appendicularia* houses (Ebersbach & Trull, 2008; Turner, 2015). However, crustaceous zooplankton will often fragment and partially consume detrital POC such as fecal pellets, as shown by laboratory experiments (Lampitt et al., 1990).

Fragmentation can be beneficial for zooplankton. Microbes rapidly colonize smaller particles (Kiørboe, 2003; Kiørboe et al., 2002), and these microbes are rich in essential nutrients (e.g., unsaturated fatty acids) (Okuyama et al., 2008; Russell & Nichols, 1999; Shulse & Allen, 2010) some of which zooplankton cannot synthesize (De Carvalho & Caramujo, 2012; Moi et al., 2018; Okuyama et al., 2008). This process of fragmentation to nurture then consume nutritionally enriched food (i.e., microbes plus detrital POC) is termed microbial gardening (Fenchel, 1970; Mayor et al., 2014). There are three key steps in microbial gardening; (a) a particle is fragmented by zooplankton increasing the particle surface area, (b) microbes colonize the newly exposed organic material rapidly (0.1–1 mm^3^ per hour, Kiørboe et al., 2003) increasing particle‐attached microbial abundance leading to increased remineralization of POC, and (c) zooplankton ingest the microbes and POC, thus their food is more nutritious than the POC without the additional microbes (Anderson et al., 2017). Fragmentation for microbial gardening is thus particularly beneficial to zooplankton in the mesopelagic zone (200–1,000 m depth) where the dominant food source is detritus (Ebersbach & Trull, 2008), which is often low in essential fatty acids that the zooplankton require (Mayor et al., 2014; Wakeham et al., 1997). However, such gardening can be seen as a gamble by choosing to fragment rather than ingest food in the food‐poor mesopelagic (Iversen, 2014).

Microbial gardening has been empirically proven in other ecosystems and habitats, for instance on detrital turtle grass fragmented by amphipods (Fenchel, 1970) and marine benthic invertebrates (Troch et al., 2005). However, the theory that fragmentation and the release of dissolved organic material further enhance microbial remineralization, beyond particle size alone, has not yet been empirically tested on sinking biogenic particles. A biogeochemical modeling study of mesopelagic carbon stocks revealed this fragmentation process was essential to balance the carbon budget of the mesopelagic zone, by routing POC through the microbial loop (Giering et al., 2014). Physical (e.g., shear) or biological fragmentation of POC has been found to account for 50% of the loss of sinking carbon with depth in the oceans (Briggs, 2020), although what proportion of this is directly linked to zooplankton particle fragmentation and microbial gardening is unknown. In addition, the lack of experimental research on this process means we do not fully understand its constraints and controls. Given that microbial gardening is a prominent example of how ecological processes can influence ocean biogeochemistry (Cavan et al., 2019), it is essential we improve our mechanistic understanding of this process.

The production of smaller particles from fragmentation can indirectly result in a feedback to atmospheric CO_2_ concentrations, by potentially increasing the surface area for microbial remineralization. Hence, ocean carbon cycle models need to be able to parameterize the decline of POC flux with depth as a function of particle size and fragmentation. There have been many studies that have investigated the role of particle size on the remineralization rate of both sinking aggregates and pellets (Belcher et al., 2016; Cavan et al., 2017; Iversen & Ploug, 2013; Ploug et al., 2008). However, these studies have employed intact pellets or those fragmented in situ prior to the onset of the experiment, and therefore, the effects of fragmentation (i.e., exposure of new POC surface areas and release of dissolved organic carbon, DOC) are dampened. Thus, studying the impact on fragmentation in a controlled laboratory setting has the potential to advance our understanding of microbial gardening and fragmentation processes. Models will also need to incorporate mesopelagic parameterizations of the energetic gains for zooplankton associated with microbial gardening, due to the increase in consumption of microbes with essential fatty acids (e.g., enhanced growth, Anderson et al., 2017). However, experiments are required first to test the hypothesis that smaller, fragmented particles are remineralized by microbes faster, and to determine in what scenario it is beneficial for zooplankton to invest in microbial gardening.

Here, we use aquaria‐maintained Antarctic krill (*Euphausia superba*) fecal pellets as a representative mesopelagic particle type (Belcher et al., 2019; Cavan et al., 2015) and measure microbial POC turnover rates on purposefully fragmented (small) and unfragmented (large) pellets. There were two types of pellets present in the krill aquaria (brown and green pellets), so the experiment was done separately on the different colored pellets, as fecal color indicates food source of the pellet POC (Wilson et al., 2008). The aim of this study is to test experimentally the first steps in microbial gardening, that fragmentation increases microbial remineralization (POC turnover) and to combine our results with the existing literature to explore the mechanisms by which microbial gardening may occur and not be a risky strategy in the open ocean. We apply a targeted experimental approach using a unique experimental facility where we could collect fresh krill pellets with knowledge of the krill diet that resulted in defecation. We hypothesize microbial POC turnover will be higher in fragmented pellets rather than unfragmented pellets, regardless of pellet color, because of the increased surface area of POC available for the microbes to degrade.

## 2 MATERIALS AND METHODS

### 2.1 Krill research aquaria

Fecal pellets from Antarctic krill (*Euphausia superba*) were collected from animals maintained in aquaria at the Australian Antarctic Division (AAD), Kingston, Tasmania. The krill are maintained in large tanks at 0.5°C. They were collected during the Antarctic summer of 2017–2018 south of 60°S in the Indian Sector of the Southern Ocean, so had been in captivity for approximately a year at the time of the experiment in January 2019. The krill were a year older though during the separate “in aquaria” fecal production and fragmentation observational study which was conducted in April 2020. The krill used were adults with an estimated age of 4–6 years. Approximately 2,000 krill were held in a 1.7 tonne circular tank. The irradiance at the time of the experiment was set to a “June” (Austral winter) regime. The krill are fed every morning, starting between 08:00 and 09:30 Australian Eastern Time (AET) with a mixture of live phytoplankton cultured at the AAD (the diatom *Phaeodactylum triconutum* and the flagellate *Pyramimonas gelidicola*, 10 L of each), 200 ml of 10% instant algae shellfish diet diluted in seawater (Reed Mariculture) and FriPack FRESH prawn hatchery feed (2.5 g diluted into 200 ml of seawater). The aquaria is a recirculating system, which continuously circulates and filters the seawater in the system. Typically, it takes about 1.5 hr for a complete exchange of the water in the tank. The water flow in the tank was closed while feeding to maximize the chance for krill to feed on the added algal mixture. See Kawaguchi et al. (2010) for more details on the aquaria setup.

### 2.2 Collection of fecal pellets

**FIGURE 1:**
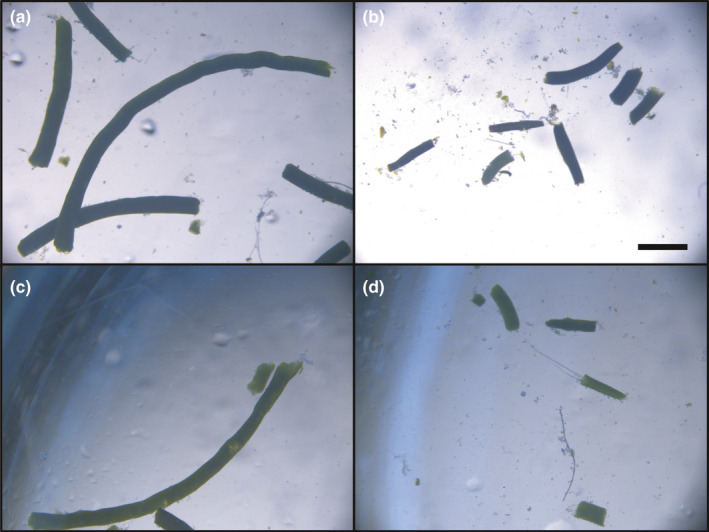
Images of different color and size fecal pellets; (a) large brown unfragmented, (b) small brown fragmented, (c) large green unfragmented, and (d) small green fragmented. The horizontal scale bar in panel (b) is 1 mm long

For the main POC turnover experiment, fecal pellet collection occurred from 09:30 to 11:30 (AET) each morning, approximately 1.5 hr after the start of feeding. As part of the aquaria protocol, pellets are removed each day from the tank. Only pellets floating in the surface waters of the tank were used for this experiment. Pellets were collected using a plastic beaker with the base cut off and replaced with 100 µm mesh, as krill pellets range from 80 to 600 µm width and 0.5 to 34 mm in length (Atkinson et al., 2012). As pellets were collected, they were rinsed from the mesh with filtered seawater into a 1‐L beaker. At least 300 pellets were collected per experiment over the 1.5 hr period and during this collection period, both green and brown pellets were present. Pellets were gently separated into two beakers depending on their color, green or brown. A subsample (~50 pellets) of each color of unfragmented pellets were then taken and stored in the fridge (4°C) for subsequent photography using light microscopy (Figure 1). Fragmented pellets (see below) were gently pipetted from the vials which had been inverted and stored in the fridge along with the larger unfragmented pellets for imaging. The majority of pellets were used in the remineralization experiment.

### 2.3 Pellet fragmentation and remineralization experiment

The aim was to compare microbial POC turnover rates on fecal pellets that had and had not been fragmented, by physically breaking recently collected krill fecal pellets. Pellets from the two beakers were transferred gently into four 20‐ml microrespiratory vials. The microrespiration vials were gently filled with the pellet and seawater sample to the top of the vial, the lid with a small capillary hole allowing the electrode to pass through was placed onto the chamber and a final drop of water added to the top of the lid to expel all air. The capped vials were then immersed within the baths filled with seawater so there could be no gaseous exchange between the vials and the surrounding water (Cavan & Boyd, 2018). Two vials were filled with brown pellets and two vials with green pellets. One vial of each colored pellet (1 × brown pellets and 1 × green pellets) was manually inverted once—quickly—to fragment the pellets. Fragmentation colored the water in the vial indicative of the release of dissolved organic material (DOM) from the fecal pellet (Figure S1). Two other 20‐ml vials were filled with water from the krill tank without pellets as control vials containing only free‐living microbes. Thus, for each experiment (and therefore per day) there were four treatments (1 × whole brown pellets, 1 × brown fragmented pellets, 1 × whole green pellets, 1 × green fragmented pellets), and 2 × control vials. The tank water is filtered at 1 µm and therefore would contain some heterotrophic bacteria smaller than this size that could attach to the pellets and decrease the oxygen concentration (Figure S2). The treatment vials (with pellets) would also potentially contain microbes released from the krill gut already attached to the pellet (Hansen & Bech, 1996). The tank water is kept at 0.5°C, UV sterilized, with ammonia removed through biofiltration to a level always below normal spectrophotometer detection limit (0.01 mg/L) and DOM was removed through a foam fractionator which generates fine bubbles in a water column skimming organic materials. All vials were placed in a rack and submerged in a water bath within a fridge (5°C), representing the Southern Ocean south of the sub‐Antarctic Front but north of 60°S (Govin et al., 2009). The selected temperature was higher than that of the live krill (0.5°C) to ensure a measurable signal in microbial respiration (Brown et al., 2004).

The experimental setup and protocol follows Cavan and Boyd (2018). An initial dissolved oxygen reading was taken using a PreSens microelectrode (limit of detection = 15 ppb, accuracy = 0.05% O_2_) as soon as the vials were placed in the fridge. The pellets were incubated in the dark and the dissolved oxygen concentration measured every 24 hr. The experiment was terminated after oxygen concentrations became <100 µM so the pellets did not have anoxic centers which can stimulate anaerobic respiration (Ploug & Bergkvist, 2015). Termination occurred after 72 hr, resulting in 4 oxygen measurements per experiment. The vials were gently (so as not to further fragment pellets) manually inverted prior to oxygen measurements to mix the water.

The experiment was replicated four times, giving four replicates per experimental treatment. At the end of each experiment, the contents of each vial were filtered through a precombusted (overnight, 400°C) QMA‐quartz filter, dried, and then stored at room temperature. The QMA filters were then prepared and processed for POC analysis; the dried filters were placed into silver cups (Elemental Microanalysis) and 20 µl of 2 N HCl Suprapur added to each cup. The cups were placed in a fuming bell overnight to remove inorganic carbon and then dried at 60°C for two days. Cups were pelleted and then C and N analyzed on a CHN analyser (Thermo Finnigan EA 1112 Series Flash Elemental Analyser). It was not possible to provide a detailed biochemical characterization of the pellets, and hence, published data on *Euphausia superba* (Tanoue et al., 1982; Tanoue & Hara, 1986) were used to help interpret our experimental results.

### 2.4 Microbial POC turnover rates

We measured the POC turnover rates of fragmented and unfragmented krill fecal pellets as a proxy for the possible increase in microbial activity associated with the fragmentation of sinking particles as suggested by microbial gardening. POC turnover rates were computed as in Cavan and Boyd (2018). Briefly, oxygen uptake rates were determined by computing the slope of the linear regression between oxygen concentration (µmol/L) and time (hr) to give oxygen uptake (µmol L^−1^ hr^−1^, Table 1 and Table S1). All slopes from vials containing pellets showed a decrease in oxygen over 72 hr (Figure S2). The slopes of the control vials (no pellets) were subtracted from the slopes of the treatment vials for that experiment to account for any oxygen uptake by free‐living microbes using carbon from nonpellet sources. Oxygen uptake rates were converted from µmol L^−1^ hr^−1^ to µmol/h by multiplying by the volume of the vials (0.02 L) as pellets only took up a small amount of total volume in the vials (see images in Figure S1). Microbial POC turnover (*k*) was calculated as a rate per day:

(1) $$k\left(d^{‐1}\right)=\text{Oxygen}\;\text{uptake}\;(\mu \text{mol}/\text{hr)}/\text{POC}\;\text{mass}\;\left(\mu \text{mol}\right)\times 24,$$

**TABLE 1:** Oxygen uptake and POC turnover for each experimental treatment vial, FP = fecal pellet. Note the oxygen uptake results are not normalized to the amount of carbon in the vials, and so POC turnover rate (O_2_ uptake normalized to POC) should be taken as the main result. See Table S1 for the full results of the linear regression fits to calculate oxygen uptake

Experiment	FP length	FP color	O_2_ uptake (µmol L^−1^ hr^−1^)	POC turnover (day^−1^)
1	Unfragmented	Brown	1.842	0.024
1	Unfragmented	Green	1.375	0.030
1	Fragmented	Brown	2.425	0.034
1	Fragmented	Green	2.288	0.051
2	Unfragmented	Brown	3.240	0.022
2	Unfragmented	Green	3.191	0.040
2	Fragmented	Brown	4.016	0.039
2	Fragmented	Green	3.567	0.048
3	Unfragmented	Brown	3.143	0.019
3	Unfragmented	Green	2.658	0.023
3	Fragmented	Brown	4.312	0.036
3	Fragmented	Green	2.041	0.011
4	Unfragmented	Brown	1.856	0.016
4	Unfragmented	Green	3.000	0.052
4	Fragmented	Brown	3.400	0.039
4	Fragmented	Green	3.188	0.032

where the POC mass has been converted from µg to µmol so the dimensions of Equation (1) and the POC turnover are correct.

### 2.5 Data and statistical analysis

To compute POC turnover rates, first the oxygen uptake rate is calculated as the slope between oxygen concentration in the vials and time (Figure S2). Rates were calculated using linear regression with the lmList function in R (Table 1 and Table S1). The number of data points were *n* = 4 for each vial as the experiment ran for 72 hr to prevent anoxic respiration which could impact our results when O_2_ concentrations reached <100 µM in the vials (Table S2). For the linear regressions, all intercept coefficients were significant (*p* < .05) and only 4 (of 16) noncontrol vial slopes were below the *p* = .05 significance threshold, two of which were *p* < .1. The *R* ^2^ values were high for all treatment vials ranging from 0.66–1.00 to 2 d.p with the majority (>60%) greater than 0.90 (Table S1). The mean *R* ^2^ across all treatments and controls was 0.90. After dividing the oxygen uptake by POC mass (Equation 1), we calculated the mean POC uptake rate per treatment (i.e., excluding controls). We did this using all data, and including those which had a slope *p*‐value above .05. The mean POC turnover rates show that unfragmented brown pellets (BL in Table S3) had lower rates compared with the other three fractions, and removing the nonsignificant slopes only minimally changed the mean unfragmented brown pellet rate from 0.020 to 0.019 day^−1^.

We used a Welch's *t* test to compare the mean POC turnover rates between the fractions (Table S4). When data that resulted in linear regression oxygen uptake slope coefficients of *p* > .1 were removed (*n* = 14, i.e., a 90% significance threshold is set rather than 95%), there was no change in the main statistical result; that the only significant difference between any two treatments in POC uptake was between un‐ and fragmented brown pellets (Table S4b). Removing all nonsignificant (at 95% significance threshold, *p* > .05, *n* = 12) linear regression oxygen uptake slope data resulted in a Welch's *t* test *p*‐value of .07 between the un‐ and fragmented brown pellets because of the now small sample size of (*n* = 2) for the brown unfragmented pellets. Nevertheless, the mean POC turnover rates for this subsample of data for fragmented brown pellets were still almost double those of the unfragmented brown pellets (Table S4c).

**FIGURE 2:**
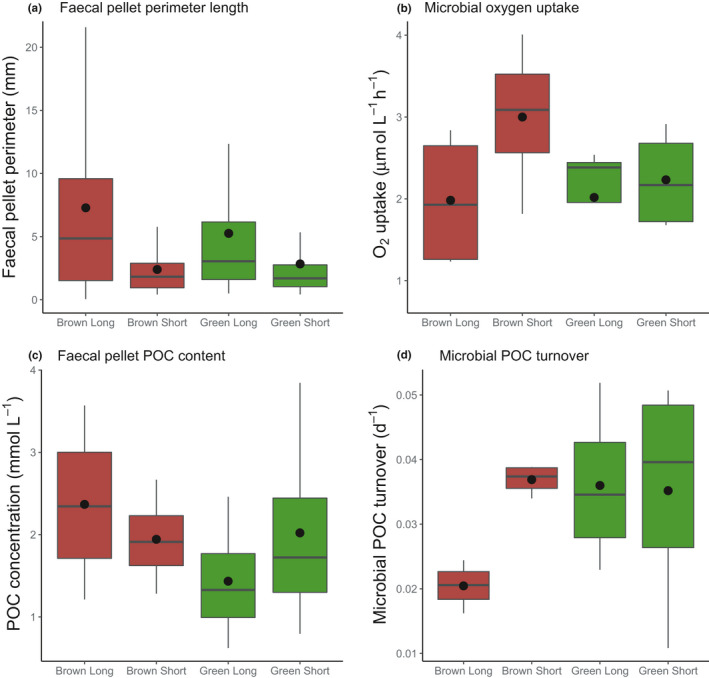
Median differences in fecal pellet size, oxygen uptake, pellet POC, and microbial POC turnover in un‐ (long) and fragmented (short) pellets of different colors (green and brown), (a) perimeter of fecal pellets (b) microbial oxygen uptake, (c) pellet POC content, and (d) microbial turnover of pellet POC. The solid black horizontal line in each box is the median, the upper and lower extent of the boxes (hinges) are the upper and lower quartiles, respectively, and the upper and lower whiskers are the highest and lowest data point. The mean is represented by the black solid symbol. The sample size is *n* = 4 for (b), (c), and (d) and *n* = 130, 278, 191, and 408, respectively, for each box in (a)

As can been seen in Figure 2d and Table S3, there was an order of magnitude more variation (standard deviation) in the green pellet POC turnover rates than both treatments of the brown pellets. As removing the few vials where the oxygen uptake slope coefficient had *p*‐values between .05 and .15 did not change the main result of our study, we present all data (*n* = 16) going forward in the results. All errors given in the main text are standard error of the mean.

### 2.6 Size of pellets

A Leica M205C dissecting stereo‐microscope with a Leica DFC 450 camera and Leica LAS V4.0 software was used to image the pellets which had been kept aside in the fridge (4°C) in darkness. Images were taken using a magnification of 32×. ImageJ was used to analyze the photographs by converting each image to 16 bit and subtracting the background with a rolling ball radius of 150 pixels. A threshold was applied and the background set to black with the pellets in white to automatically detect particles and measure their size (here perimeter), with a minimum detection limit of 0.01 mm.

### 2.7 Fecal pellet production and fragmentation by aquaria Antarctic krill

To determine how fecal pellet color and fragmentation change within the krill tank, we ran a separate experiment observing the alteration of the size and color of fecal pellets produced as the amount of food changed with time since the start of the daily morning feed. Initially, tank water was thoroughly inspected to ensure there were no fecal pellets existing prior to feeding. In this experiment, feeding started at 09:15. Water flow was closed between 09:30 and 12:30 to ensure food was not cleared out from the tank during this period. At 12:30, the water flow was turned on, decreasing the algal food supply available to the krill. Given that it takes 1.5 hr for the water to fully circulate, no algal food remained in the tank by 14:00. Sampling of fecal pellets was undertaken at 10:15, 11:15, 12:35, 14:30, and 16:45. Fecal pellets floating in the water were gently siphoned out into a bucket prior to being photographed and analyzed as above. 40–90 pellets were collected at each sampling time point, with collected pellets increasing in number with time due to fragmentation.

## 3 RESULTS

Manual fragmentation of krill fecal pellets successfully created long and short pellets as a proxy of particles of different sizes with different surface area to volume ratio in the oceans (Figures 1 and 2a, Figure S3). The mean perimeter length of unfragmented brown and green pellets was 7.28 ± 0.06 mm and 5.26 ± 0.03 mm, respectively. Both types (brown and green) of unfragmented pellets were significantly longer than the short, manually fragmented pellets (*t* test, *p* < .05, Figure 2a). The mean perimeter length of physically fragmented brown and green pellets was 2.40 ± 0.01 mm and 2.84 ± 0.01 mm, respectively. There was no statistical difference in length between the brown and green fragmented pellets (*p* > .05).

Linear regressions between oxygen concentration and time showed oxygen concentrations declined significantly (*p* < .05) over the 72‐hr experiment in each microrespiration vial which contained pellets (Figure S2, Table S1). Oxygen concentrations approached 100 µM after 72 hr in 3 of the 4 replicate experiments (Tables S1 and S2). Microbial oxygen uptake rates ranged from 1.8 to 4.3 µmol L^−1^ hr^−1^ (Table 1). Mean oxygen uptake rates per pellet treatment (color and fragmentation, Figure 2b) ranged from 2.0 to 3.0 µmol L^−1^ hr^−1^ (Figure 2b). Oxygen uptake rates do not account for the varying number of pellets (and therefore POC in each vial), and thus, the normalized values of POC turnover (Equation 1) are the focus our results (Table 1). Mean pellet POC concentration ranged from 1.4 ± 0.2 mmol/L in the unfragmented green pellets to 2.4 ± 0.3 mmol/L in the unfragmented brown pellets (Figure 2c).

After normalization of the oxygen uptake rate to fecal pellet POC, there was no overall effect of pellet size (*p* > .05, Figure 2d) on microbial POC turnover rate, as the POC turnover was the same for un‐ and fragmented green pellets and fragmented brown pellets (mean ~0.036 ± 0.01 day^−1^). However, POC turnover in the fragmented brown pellets was significantly (*p* < .05, *t* test, Table S4a) higher (1.9×) than that of unfragmented brown pellets (0.020 ± 0.00 day^−1^). Even though the difference in POC turnover rate between unfragmented and green pellets and unfragmented brown pellets was the same as between un‐ and fragmented brown pellets, the larger variation in POC turnover of green pellets (Table S3) means this difference was not significant (Table S4a). Therefore, microbial remineralization did increase upon fragmentation but only for brown pellets and not green pellets.

**FIGURE 3:**
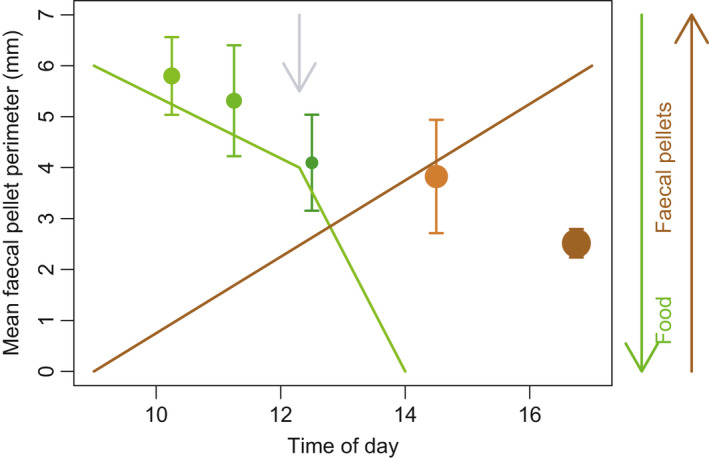
Results of the in‐tank krill time‐series experiment of fecal pellet size (perimeter) throughout the day postfeeding. The mean fecal pellet perimeters (*y*‐axis) are shown by the symbols, with error bars representing the standard error of the mean. The size of the symbols is the number of pellets in the sample collected (not normalized to volume) ranging from 40 to 91 pellets. The colors of the symbols reflect the gradual change in the dominance of green to brown pellets as observed by eye during the experiment. Broad trends in algal food concentration and fecal pellet number are indicated to show when they increased and decreased, respectively. In this experiment, feeding started at 09:15 in the morning and the water flow was turned on at 12:30 (gray downward arrow), resulting in sharp decline in algal food which was completely removed from the tank by 14:00. Fecal pellets were present in the tank at all sampling times

To find when green versus brown pellets are produced by the krill within the tank, and if in situ fragmentation occurs we ran a separate time‐series experiment. Our results indicated that while algal food was present in the tank (09:15 to 14:00, Figure 3) pellets were predominantly green. Once the tank was empty of algal food (at 14:00), the pellets became browner in color indicating the krill had begun to feed on the pellets (coprophagy) (Lampitt et al., 1990), and the organic material had now passed through the krill gut at least twice (Figure 3). We quantified the mean perimeter of these pellets, which was largest at the first time point and decreased continually throughout the day (Figure 3) indicating that either brown pellets are smaller when egested or that krill preferentially fragment brown pellets while feeding. The number of pellets in each sample taken also increased with time, so there were more pellets that were also smaller toward the end of the day. This supports the latter theory that krill were preferentially fragmenting the brown pellets. The results of this experiment provide evidence that the green pellets in this experiment had likely only passed through the krill gut once (Fuentes et al., 2016) and the brown pellets were formed from feeding on green pellets. It also suggests Antarctic krill in the tank are undertaking microbial gardening of the more detrital pellets, because of the decrease in size (Figure 3) of the browner pellets toward the end of the day. This is evidence of fragmentation as unfragmented brown pellets were not found to be smaller than green pellets in our main study prior to manual fragmentation (Figure 2a), when collected a short time (1.5 hr) after feeding.

Our results are a step toward supporting the first tenet of the microbial gardening hypothesis as they confirm that fragmentation can enhance microbial remineralization of POC and thus potentially boost the nutritional value of food consumed by mesopelagic krill or zooplankton. However, given that pellet color influenced the experimental outcome, modeling microbial gardening may not be as straightforward as we suggested.

## 4 DISCUSSION

Fragmentation in conjunction with microbial gardening has been proposed by modeling studies (Giering et al., 2014; Mayor et al., 2014) as an important ecological process in the food‐limited mesopelagic zone influencing the open‐ocean carbon cycle. Gardening may provide energy‐rich food for zooplankton, while also having biogeochemical implications by increasing the remineralization rates of sinking particulate carbon, ultimately reducing the ocean carbon sink. For the first time, we experimentally tested the first step in the microbial gardening hypothesis that fragmentation of sinking particles increases microbial activity and the dissolution of POC associated with smaller particles, using krill fecal pellets as a representative detrital particle. Our pellet POC turnover rates (0.011–0.052 day^−1^) were of the same order of magnitude as those measured from krill pellets collected from the Scotia Sea (0.05–0.065 day^−1^) (Belcher et al., 2016), but lower than those measured on copepod pellets produced in the laboratory (0.08–0.21 day^−1^) (Ploug et al., 2008) or collected in situ (0.52–2.5 day^−1^) (Poulsen & Iversen, 2008). The lower POC turnover rates on krill pellets compared with copepods is likely due to temperature of the experiments (Brown et al., 2004; Cavan & Boyd, 2018), as in both copepod studies most of the treatments were run at higher temperatures than our (5°C) experiment, 4–16°C (Poulsen & Iversen, 2008), and 15°C (Ploug et al., 2008).

**FIGURE 4:**
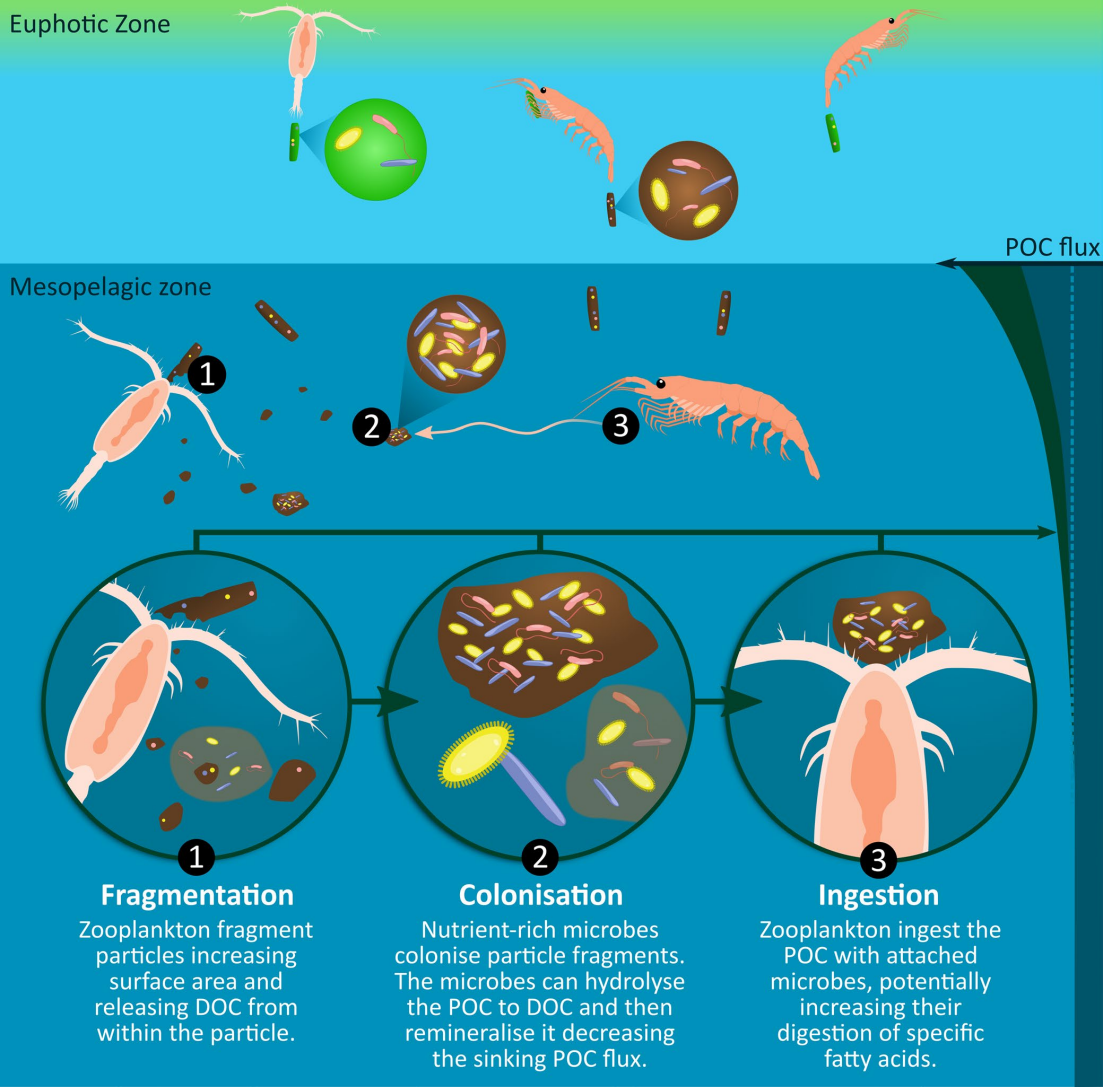
In the euphotic zone zooplankton and krill feed on phytoplankton cells, producing greener fecal pellets containing more labile organic matter with some essential nutrients. As these pellets or particles sink and are consumed (Lampitt et al., 1990), the highly nutritious compounds are preferentially removed, such that within the underlying mesopelagic zone food is scarcer and less nutritious, and pellets are brown in color. To gain essential nutrients mesopelagic resident or migratory zooplankton they (1) fragment detrital food reducing the size of the particles and releasing dissolved organic carbon (DOC), which (2) promotes the colonization of microbes many of which can synthesize nutrients essential to zooplankton (e.g., unsaturated lipids, see Shulse & Allen, 2011 and Yoshida et al., 2016), and (3) the zooplankton ingest both the particle and the attached microbes, which now likely contains additional nutrients zooplankton need (see Tanoue et al., 1982). Under the communal gardening concept, zooplankton may garden and consume the entire particle and many colonized bacteria, or may move on leaving the particle and colonizers for other grazers, they themselves benefiting from other abandoned particles. The reduced size and therefore sinking rate of the fragmented particles, and the increased remineralization by particle‐attached (and free‐living on any DOC released via fragmentation) microbes are responsible for large decreases in the sinking POC flux

Our results showed that small pellets are subjected to higher remineralization rates than larger pellets, but only when the pellets were brown in color, and thus particle size alone is not a unique driver of remineralization rates. Sinking fecal pellets collected from the ocean, including those of krill, tend to be brown or white depending on diet (Wassmann et al., 1994; Werner, 2000; Wilson et al., 2008), with whiter pellets being from those feeding on detrital food sources which are less nutritious. In our aquaria setting at the AAD, the krill only ever produce green or brown pellets. Here, the krill are the same age and species, so diet most likely contributes to the difference in pellet color (Werner, 2000). In the aquaria fragmentation experiment, green pellets were produced after a supply of fresh phytoplankton cells to the tank (Figure 3), and as the digestive tract of Antarctic krill is green when feeding on phytoplankton (Fuentes et al., 2016), the green pellets collected in this study for the remineralization experiments were from those feeding on phytoplankton. As green pellets are not commonly observed in the ocean, particularly in sediment traps, they are likely preferentially consumed in the surface ocean and thus are more indicative of euphotic zone food sources (Figure 4). The brown pellets were mostly present in our aquaria fragmentation experiment once the phytoplankton food source had been depleted, and thus, we propose that brown pellets occur due to krill feeding on other pellets (coprophagy, Lampitt et al., 1990) when fresh algal concentrations are low (Figure 3) as is the case in the mesopelagic (Figure 4).

Pellets are first observed in the AAD aquarium tanks 20–30 min after the krill commence feeding, consistent with laboratory experiments of krill gut clearance times (Pond et al., 1995). Thus, in our study there was sufficient time for both green and brown pellets to be present when pellets were collected for the POC turnover rate experiment. These brown pellets would likely contain more refractory material than the green pellets, although we did not characterize their biochemical composition in our study. From the literature, most of the essential nutrients and labile organic carbon, such as unsaturated fatty acids and amino acids, have already been extracted from such refractory material leaving compounds that are less nutritious to consumers (Zimmerman & Canuel, 2001). This conclusion is supported by time‐series experiments conducted by Neal et al. (1986) and Harvey et al. (1991) on the lipid composition of fecal pellets. Both studies report that feeding mode (i.e., herbivory vs. cophrophagy) had a major influence on proportion of refractory material in the pellets (cophrophagy resulted in more refractory pellets). This trend is also evident for Antarctic krill – Pond et al. (1995) fed them with radio‐labeled phytoplankton and analyzed assimilation and egestion of algal lipids. Their polar study demonstrates that the hydrocarbon detected in the algal lipid was assimilated efficiently and eicosapentaenoic acid (EPA) was catabolized extensively (65%–93%). Both unfragmented and fragmented green pellets (i.e., with more labile, energy‐rich organic carbon) were turned over at the same rate showing that if the carbon contains high energy compounds, the microbes will remineralize the carbon at the same rate regardless of particle size. As only the fragmentation of brown pellets increased POC turnover, we conclude that when the organic material is energy‐poor and microbes are substrate limited, fragmentation facilitates the exposure of new material for microbes to remineralize.

Many different microbes can colonize organic particles and contribute to both trophic upgrading (i.e., increasing the proportion of essential nutrients) of the organic material and POC degradation and remineralization of DOC. Trophic upgrading is viewed as the main benefit of microbial gardening to zooplankton (Anderson et al., 2017; Mayor et al., 2014) as the micron‐scale size, mass and carbon content of microbes means they do not increase the magnitude of food items for zooplankton. This is readily demonstrated—if we assume bacteria occupy 10% of the surface area of fecal pellets after incubation (Gowing & Silver, 1983), have a carbon content per cell of 12 fg C/cell (see Boyd et al., 2015) and are 2 µm diameter, and krill pellets have 0.39 µg C/pellet (Gleiber et al., 2012) and a pellet surface area of 5.5 mm^2^ (converting of a perimeter of 6 mm to a cuboid of dimensions 0.5 × 0.5 × 2.5 mm), then bacterial colonization would only contribute to 0.54% of pellet POC. It is more likely the microbes actually decrease the POC through solubilization and remineralization. Many pellet degradation studies have found that other planktonic groups aside from bacteria, such as dinoflagellates and ciliates can be the key degraders of pellet POC (Jacobsen & Azam, 1984; Poulsen & Iversen, 2008), while others reported that bacteria can significantly contribute to pellet degradation (Jing et al., 2012). Regardless, free‐living bacteria remineralize the DOC released upon fragmentation by the pellet (Thor & Dam, 2003). Microbes, including dinoflagellates and bacteria, may also increase the nutritional value of detrital food (Anderson et al., 2017), which we now explore.

How do we reconcile the main outcome of our study—that fragmented brown (i.e., refractory) pellets have higher carbon turnover than for intact brown fecal pellets—with the likelihood that enhanced bacterial activity, promoted by gardening via fragmentation, will not result in any significant increase in the POC content of the pellet (0.54%)? If microbial gardening is taking place then there must be some benefits other than the enhancement of pellet POC.

Zooplankton can synthesize some but not all essential lipids or amino acids so they must derive some from their food source, otherwise they may be susceptible to poor growth and fecundity and high mortality (Stoecker & Capuzzo, 1990). Many marine bacteria can synthesize two essential polyunsaturated lipids, eicosapentaenoic acid (EPA) and docosahexaenoic acid (DHA) (Russell & Nichols, 1999; Shulse & Allen, 2010, 2011), which make up 30% of total fatty acids in bacteria (Okuyama et al., 2008). Particle‐attached bacteria attract microbial predators (see supplementary video in Boyd et al., 2017) such as dinoflagellates, which can synthesize DHA, potentially further upgrading the nutritional quality of the pellet as a food source for zooplankton (Klein Breteler et al., 1999). Thus, colonizing microbes make up a much higher proportion of these lipids as a percentage of all the fatty acids, than in sinking detrital mesopelagic POM (4% for EPA and 10% for DHA) (Mayzaud et al., 2014). In contrast, fatty acids associated with mesopelagic POM are comprised of more refractory saturated fatty acids (Cavan et al., 2018; Wakeham et al., 1997).

In the case of the fecal pellets of Antarctic krill, EPA comprised 2.3% by weight (c.f. 0.7% and 2.0% for POM and net plankton samples, respectively) (DHA was not assayed, Tanoue & Hara, 1986). This outcome differs from experiments conducted with the pellets from temperate copepods in which the abundance of heterotrophic bacteria increased over time, as did poly unsaturated fatty acids but not EPA and DHA, which were completely removed (Harvey et al., 1991; Neal et al., 1986). Clearly, more research is needed into changes with depth (and time) in the biochemical characteristics of krill pellets, along with the role that microbes play in nutritional enhancement of the pellets. There must be potential mechanisms to supply EPA to animals at depth (Tanoue & Hara, 1986), where Antarctic krill often feed (Clarke & Tyler, 2008; Schmidt et al., 2011) and/or reproduce (Kawaguchi et al., 2011).

Our experiment suggested higher microbial activity on brown pellets of lower nutritional value than green pellets, thus nutritionally poor food is more likely to benefit from trophic upgrading and the addition of extra essential fatty acids. As green pellets are more indicative of euphotic zone food sources (Figure 3), green pellets may be consumed in surface waters and be subsequently egested as brown pellets which sink into the mesopelagic, which are then actively fragmented (Figure 4). A laboratory study where copepods have the choice between pellets and an alternative food source show pellets were mostly rejected and fragmented by the copepods as they preferentially chose the more nutritious food (Iversen & Poulsen, 2007). However as in the mesopelagic zone the food source is predominantly detrital (Ebersbach & Trull, 2008), pellet and detrital ingestion is higher (Suzuki et al., 2003) than in surface waters. Zooplankton may be able to detect the nutritional value of the particles they intercept (Friedman & Strickler, 1975; Kiørboe & Jackson, 2001) and actively chose whether to fragment them. Copepods can use chemo‐detection to find particles (Jackson & Kiørboe, 2004), and zooplankton are known to select food based on nutritional value (DeMott, 1990; Vanderploeg, 1994). Ultimately, the time for which a zooplankter remains in contact with the particles it has fragmented and consume microbes will be influenced by turbulence.

Turbulence is low in the mesopelagic compared with the surface ocean with the smallest turbulent eddies (10 × Kolmogorov length scale) being larger (10 mm flow length scale) (Siegel, 1998) than even unfragmented pellets in this study (<6 mm mean perimeter length), laboratory produced aggregates (<7 mm equivalent spherical diameter) (Iversen & Ploug, 2013) and natural sinking particles observed in the ocean (<3 mm equivalent spherical diameter) (Cavan et al., 2018). Fragmented particles will be smaller than the length scale of turbulent eddies in the mesopelagic (Figures 2a and 3). Thus, it is reasonable to predict that in the less turbulent mesopelagic zone a zooplankton may remain in the vicinity of its fragmented particle for some time, thus making it worthwhile to expend energy fragmenting particles and consuming the fragments and microbes as they attach for some time postfragmentation. On the contrary, zooplankton may not stay with the particle long enough for microbial colonization to take place. Thus, “communal gardening” may be a more plausible scenario, whereby zooplankton partially consume labile material from their fragmented particle before moving onto other food sources, meanwhile the particle is continuously colonized by microbes which a neighboring zooplankton or one living in underlying waters may then ingest (Figure 4).

There is circumstantial evidence of such a communal approach. Mesopelagic copepods living in the mesopelagic below sea ice in the Southern Ocean have been shown to fragment krill fecal pellets, which then can be a food source for deeper living organisms (Suzuki et al., 2003). Communal gardening may be particularly beneficial for large aggregations of zooplankton such as krill swarms (Tarling & Fielding, 2016) and other zooplankton and fish living in deep scattering layers (Proud et al., 2017). Diel vertical migrators may have further advantage from accessing and grazing both phytoplankton in the euphotic zone and to gardened particles in the mesopelagic zone.

Although microbial gardening has been proven to enhance the nutritional value of detrital food for estuarine copepods (Heinle et al., 1977), it can seem an odd and potentially controversial choice for mesopelagic organisms where food is scarce. Why would a zooplankton pass up the opportunity for food and instead fragment the particle? The modeling study of Mayor et al. (2014) proposing microbial gardening in the mesopelagic zone is supported with video evidence of preferential ingestion of small pellets over large ones by copepods (Iversen, 2014; Iversen & Poulsen, 2007). A more recent study has shown in the North Pacific that small particles (<53 µm) dominated the diet of mesopelagic zooplankton when particle flux (i.e., their food source) was low (Romero‐Romero et al., 2020), further supporting the evidence that fragmentation of particles is common in the mesopelagic zone, with consequences for the carbon cycle (Briggs, 2020).

As previously mentioned, a side effect of microbial gardening is increased remineralization to CO_2_ or solubilization to dissolved organic carbon (DOC) of POC by particle‐ or pellet‐attached bacteria. Increased remineralization by free‐living bacteria will also occur on any DOC released from inside a particle (Figure 4 and Figure S1). As the fragmented particles are smaller, they will also sink more slowly (Iversen & Ploug, 2013; Laurenceau‐Cornec et al., 2019). The increased microbial activity and reduced sinking rates decreases the sink of POC in the ocean, reducing the capacity of deep ocean storage of atmospheric CO_2_. Fragmentation of particles as they sink and subsequent remineralization by microbes is responsible for 50% of the decline in POC with depth (Briggs, 2020). This includes both physical fragmentation of more fragile aggregates and biological fragmentation by zooplankton. Thus, an ecological process to gain essential nutrients (microbial gardening) has wider implications on the carbon cycle and biogeochemistry. Linking ecological and biogeochemical processes are important to understand ecosystem feedbacks on climate (Cavan et al., 2019; Schmitz et al., 2013) including in modeling work. Here, we have shown that particle size alone is not a good enough descriptor of microbial activity and remineralization, as particle food source and composition are important constraining factors.

Before being able to parameterize microbial gardening in carbon models, future experiments and observations should determine the extent of fragmentation in different food environments for zooplankton such as replete and depleted food sources and those of different labilities, as inferred by organic chemistry, simulating the euphotic versus mesopelagic zone. While fecal pellets are an important component of particle flux globally, a similar empirical study to this is needed on phytodetrital aggregates, to show if particle composition influences the remineralization rate of different sized aggregates in the same way it does fecal pellets. Information is also required on the nutritional and energetic gains of microbial gardening to zooplankton, and how this may impact carbon transfer efficiency and carbon cycling, and how remineralization rates may differ if fragmentation occurs through physical shear or is biologically mediated. Further research is also important to predict or forecast remineralization rates with climate change. As most models parameterize remineralization as a function of temperature or oxygen, research is required on how the impacts of particle size and lability on remineralization compare to these physical controls. It is likely that resident mesopelagic zooplankton (i.e., those not undergoing diel vertical migration to feed at the surface) will benefit most from fragmenting particles they intercept, and hence euphotic zone models may need not incorporate microbial gardening processes.

## 5 CONCLUSIONS

We present experimental evidence toward refining the microbial gardening hypothesis by mesopelagic zooplankton. Previously modeling studies have shown that fragmentation of sinking organic particles by zooplankton can account for a large loss of the ocean carbon sink due to the increase in microbial remineralization on the smaller particles. We suggest though that zooplankton only fragment more refractory particles typically found in the mesopelagic zone, as only brown pellets in this study showed an increase in microbial activity when fragmented. Hence, particle composition is an important factor, with more detrital particles likely to be fragmented. As zooplankton can detect the nutritional value of the food they encounter they may actively choose to fragment the sinking particle or not. The ecological benefit of fragmenting a particle rather than consuming it is that many microbes, composed of essential nutrients required by zooplankton, colonize these smaller particles, which the zooplankton can then ingest. More information is required on how microbes alter the biochemical characteristics of krill pellets with depth (and time), since they have the potential to supply EPA to krill at depth where they often feed and/or reproduce. Other tenets of microbial gardening that require more research is whether zooplankton remain within close proximity long enough to benefit from substantial bacterial colonization, or abandon the fragments after initial consumption of labile compounds released. The latter hypothesis suggests a level of altruism among mesopelagic communities. Regardless, the increased abundance of microbes on smaller particles increases the remineralization of organic carbon, declining the ocean carbon sink. Hence, microbial gardening is an example of the impacts of ecology on biogeochemistry. It is clear from this and previous studies that microbial gardening is an important process in the mesopelagic zone, and one that is worthy of parameterizations in biogeochemical models. However, prior to that, additional and extended experimental work is needed to investigate the controls and conditions of this process.

## CONFLICT OF INTEREST

The authors state no competing interests.

## AUTHOR CONTRIBUTIONS

**Emma L. Cavan:** Conceptualization (lead); Formal analysis (lead); Investigation (lead); Methodology (lead); Visualization (lead); Writing‐original draft (lead); Writing‐review & editing (lead). **Philip W. Boyd:** Conceptualization (equal); Writing‐review & editing (equal). **So Kawaguchi:** Investigation (equal); Methodology (equal); Resources (equal); Writing‐review & editing (equal).

## Supporting information

Supplementary Material

## Data Availability

Data are available at https://doi.org/10.5061/dryad.r7sqv9s9x and the R code to reproduce the figures at https://github.com/e-cavan/microbial_gardening_krill.

## References

[ece37119-bib-0001] Anderson, T. R. , Pond, D. W. , & Mayor, D. J. (2017). The role of microbes in the nutrition of detritivorous invertebrates: A stoichiometric analysis. Frontiers in Microbiology, 7, 1–13. 10.3389/fmicb.2016.02113 PMC5209341 28101083

[ece37119-bib-0002] Atkinson, A. , Schmidt, K. , Fielding, S. , Kawaguchi, S. , & Geissler, P. A. (2012). Variable food absorption by Antarctic krill: Relationships between diet, egestion rate and the composition and sinking rates of their fecal pellets. Deep‐Sea Research Part II: Topical Studies in Oceanography, 59–60, 147–158. 10.1016/j.dsr2.2011.06.008

[ece37119-bib-0003] Belcher, A. , Henson, S. A. , Manno, C. , Hill, S. L. , Atkinson, A. , Thorpe, S. E. , Fretwell, P. , Ireland, L. , & Tarling, G. A. (2019). Krill faecal pellets drive hidden pulses of particulate organic carbon in the marginal ice zone. Nature Communications, 10, 889. 10.1038/s41467-019-08847-1 PMC6385259 30792498

[ece37119-bib-0004] Belcher, A. , Iversen, M. , Manno, C. , Henson, S. A. , Tarling, G. A. , & Sanders, R. (2016). The role of particle associated microbes in remineralization of fecal pellets in the upper mesopelagic of the Scotia Sea, Antarctica. Limnology and Oceanography, 61, 1049–1064.

[ece37119-bib-0005] Boyd, P. W. , Ellwood, M. J. , Tagliabue, A. , & Twining, B. S. (2017). Biotic and abiotic retention, recycling and remineralization of metals in the ocean. Nature Geoscience, 10, 167–173. 10.1038/ngeo2876

[ece37119-bib-0006] Boyd, P. W. , Strzepek, R. F. , Ellwood, M. J. , Hutchins, D. A. , Nodder, S. D. , Twining, B. S. , & Wilhelm, S. W. (2015). Why are biotic iron pools uniform across high‐ and low‐iron pelagic ecosystems? Global Biogeochemical Cycles, 29, 1028–1043. 10.1002/2014GB005014

[ece37119-bib-0007] Briggs, N. , Dall’Olmo, G. , & Claustre, H. (2020). Major role of particle fragmentation in regulating biological sequestration of CO_2_ by the Oceans. Science, 793, 791–793. 10.1126/science.aay1790 32054763

[ece37119-bib-0008] Brown, J. H. , Gillooly, J. F. , Allen, A. P. , Savage, V. M. , & West, G. B. (2004). Toward a metbolic theory of ecology. Ecology, 85, 1771–1789.

[ece37119-bib-0009] Cavan, E. L. , Belcher, A. , Atkinson, A. , Hill, S. L. , Kawaguchi, S. , McCormack, S. , Meyer, B. , Nicol, S. , Ratnarajah, L. , Schmidt, K. , Steinberg, D. K. , Tarling, G. A. , & Boyd, P. W. (2019). The importance of Antarctic krill in biogeochemical cycles. Nature Communications, 10, 1–13. 10.1038/s41467-019-12668-7 PMC6800442 31628346

[ece37119-bib-0010] Cavan, E. L. , & Boyd, P. W. (2018). The effect of anthropogenic warming on microbial respiration and particulate organic carbon export rates in the sub‐Antarctic Southern Ocean. Aquatic Microbial Ecology, 82, 111–127.

[ece37119-bib-0011] Cavan, E. L. , Giering, S. L. C. , Wolff, G. A. , Trimmer, M. , & Sanders, R. (2018). Alternative particle formation pathways in the eastern tropical north Pacific's biological carbon pump. Journal of Geophysical Research: Biogeosciences, 123, 2198–2211.

[ece37119-bib-0012] Cavan, E. L. , Le Moigne, F. A. C. , Poulton, A. J. , Tarling, G. A. , Ward, P. , Daniels, C. J. , Fragoso, G. M. , et al. (2015). Attenuation of particulate organic carbon flux in the Scotia Sea, Southern Ocean, is controlled by zooplankton fecal pellets. Geophysical Research Letters, 42, 821–830.

[ece37119-bib-0013] Cavan, E. L. , Trimmer, M. , Shelley, F. , & Sanders, R. (2017). Remineralization of particulate organic carbon in an ocean oxygen minimum zone. Nature Communications, 8, 1–9. 10.1038/ncomms14847 PMC5364423 28322218

[ece37119-bib-0014] Clarke, A. , & Tyler, P. A. (2008). Adult Antarctic krill feeding at Abyssal Depths. Current Biology, 18, 282–285. 10.1016/j.cub.2008.01.059 18302926

[ece37119-bib-0015] De Carvalho, C. C. C. R. , & Caramujo, M. J. (2012). Lipids of prokaryotic origin at the base of marine food webs. Marine Drugs, 10, 2698–2714. 23342392 10.3390/md10122698 PMC3528120

[ece37119-bib-0016] DeMott, W. R. (1990). Retention efficiency, perceptual bias, and active choice as mechanisms of food selection by suspension‐feeding zooplankton In Behavioural mechanisms of food selection (pp. 569–594). Springer.

[ece37119-bib-0017] Ebersbach, F. , & Trull, T. W. (2008). Sinking particle properties from polyacrylamide gels during the KErguelen Ocean and Plateau compared Study (KEOPS): Zooplankton control of carbon export in an area of persistent natural iron inputs in the Southern Ocean. Limnology and Oceanography, 53, 212–224. 10.4319/lo.2008.53.1.0212

[ece37119-bib-0018] Fenchel, T. (1970). Studies on the decomposition of organic detritus derived from the turtle grass *Thalassia testudinum* . Limnology and Oceanography, 15, 14–20.

[ece37119-bib-0019] Friedman, M. M. , & Strickler, J. R. (1975). Chemoreceptors and feeding in calanoid copepods (Arthropoda: Crustacea). Proceedings of the National Academy of Sciences USA, 72, 4185–4188. http://www.pnas.org/content/72/10/4185.abstract 10.1073/pnas.72.10.4185 PMC433165 1060099

[ece37119-bib-0020] Fuentes, V. , Alurralde, G. , Meyer, B. , Aguirre, G. E. , Canepa, A. , Wölfl, A.‐C. , Hass, H. C. , et al. (2016). Glacial melting: An overlooked threat to Antarctic krill. Scientific Reports, 6, 27234 10.1038/srep27234 27250339 PMC4890292

[ece37119-bib-0021] Giering, S. , Sanders, R. , Lampitt, R. , Anderson, T. , Tamburini, C. , Boutrif, M. , Zubkov, M. , et al. (2014). Reconciliation of the carbon budget in the ocean's twilight zone. Nature, 507, 480–483. 24670767 10.1038/nature13123

[ece37119-bib-0022] Gleiber, M. R. , Steinberg, D. K. , & Ducklow, H. W. (2012). Time series of vertical flux of zooplankton fecal pellets on the continental shelf of the western Antarctic Peninsula. Marine Ecology Progress Series, 471, 23–36.

[ece37119-bib-0023] Govin, A. , Michel, E. , Labeyrie, L. , Waelbroeck, C. , Dewilde, F. , & Jansen, E. (2009). Evidence for northward expansion of Antarctic bottom water mass in the Southern Ocean during the last glacial inception. Paleoceanography and Paleoclimatology, 24(1).

[ece37119-bib-0024] Gowing, M. M. , & Silver, M. W. (1983). Origins and microenvironments of bacteria mediating fecal pellet decomposition in the sea. Marine Biology, 73, 7–16. 10.1007/BF00396280

[ece37119-bib-0025] Hansen, B. , & Bech, G. (1996). Bacteria associated with a marine planktonic copepod in culture. I. Bacterial genera in seawater, body surface, intestines and fecal pellets and succession during fecal pellet degradation. Journal of Plankton Research, 18, 257–273.

[ece37119-bib-0026] Harvey, H. R. , Eglinton, G. , O'Hara, S. C. M. , & Corner, E. D. S. (1991). Microbial transformation of fecal pellet lipids during sedimentation. Developments in Geochemistry, 6, 25–36.

[ece37119-bib-0027] Heinle, D. R. , Harris, R. P. , Ustach, J. F. , & Flemer, D. A. (1977). Detritus as food for estuarine copepods. Marine Biology, 40, 341–353. 10.1007/BF00395727

[ece37119-bib-0028] Iversen, M. H. (2014). Is microbial gardening a food gamble or a safe bet? (Comment on DOI 10.1002/bies.201400100). BioEssays, 36: 1126 10.1002/bies.201400181 25382778

[ece37119-bib-0029] Iversen, M. H. , & Ploug, H. (2013). Temperature effects on carbon‐specific respiration rate and sinking velocity of diatom aggregates – potential implications for deep ocean export processes. Biogeosciences, 10, 4073–4085.

[ece37119-bib-0030] Iversen, M. , & Poulsen, L. (2007). Coprorhexy, coprophagy, and coprochaly in the copepods *Calanus helgolandicus*, *Pseudocalanus elongatus*, and *Oithona similis* . Marine Ecology Progress Series, 350, 79–89.

[ece37119-bib-0031] Jackson, G. A. , & Kiørboe, T. (2004). Zooplankton use of chemodetection to find and eat particles. Marine Ecology Progress Series, 269, 153–162.

[ece37119-bib-0032] Jacobsen, T. R. , & Azam, F. (1984). Role of bacteria in copepod fecal pellet decomposition: colonization, growth rates and mineralization. Bulletin of Marine Science, 35, 495–502.

[ece37119-bib-0033] Jing, H. , Shek, L. , Yung, W. , Jin, X. , & Liu, H. (2012). Dynamics of bacterial community composition during degradation of copepod fecal pellets. Journal of Plankton Research, 34, 700–710.

[ece37119-bib-0034] Kawaguchi, S. , Kilpatrick, R. , Roberts, L. , King, R. , & Nichol, S. (2011). Ocean‐bottom krill sex. Journal of Plankton Research, 33, 1134–1138. 21655471 10.1093/plankt/fbr006 PMC3109991

[ece37119-bib-0035] Kawaguchi, S. , King, R. , Meijers, R. , Osborn, J. E. , Swadling, K. M. , Ritz, D. A. , & Nicol, S. (2010). An experimental aquarium for observing the schooling behaviour of Antarctic krill (*Euphausia superba*). Deep Sea Research Part II: Topical Studies in Oceanography, 57, 683–692.

[ece37119-bib-0036] Kiørboe, T. (2003). Marine snow microbial communities: Scaling of abundances with aggregate size. Aquatic Microbial Ecology, 33, 67–75.

[ece37119-bib-0037] Kiørboe, T. , Grossart, H. P. , Ploug, H. , & Tang, K. (2002). Mechanisms and rates of bacterial colonization of sinking aggregates. Applied and Environmental Microbiology, 68, 3996–4006. 12147501 10.1128/AEM.68.8.3996-4006.2002 PMC124032

[ece37119-bib-0038] Kiørboe, T. , & Jackson, G. A. (2001). Marine snow, organic solute plumes, and optimal chemosensory behavior of bacteria. Limnology and Oceanography, 46, 1309–1318. 10.4319/lo.2001.46.6.1309

[ece37119-bib-0039] Kiørboe, T. , Tang, K. , Grossart, H.‐P. , & Ploug, H. (2003). Dynamics of microbial communities on marine snow aggregates: Colonization, growth, detachment, and grazing mortality of attached bacteria. Applied and Environmental Microbiology, 69, 3036–3047. 12788697 10.1128/AEM.69.6.3036-3047.2003 PMC161531

[ece37119-bib-0040] Klein Breteler, W. C. M. , Schogt, N. , Baas, M. , Schouten, S. , & Kraay, G. W. (1999). Trophic upgrading of food quality by protozoans enhancing copepod growth: Role of essential lipids. Marine Biology, 135, 191–198. 10.1007/s002270050616

[ece37119-bib-0041] Lampitt, R. , Noji, T. , & Bodungen, B. (1990). What happens to zooplankton faecal pellets? Implications for vertical flux. Marine Biology, 23, 15–23.

[ece37119-bib-0042] Laurenceau‐Cornec, E. C. , Le Moigne, F. A. C. , Gallinari, M. , Moriceau, B. , Toullec, J. , Iversen, M. H. , Engel, A. , et al. (2019). New guidelines for the application of Stokes' models to the sinking velocity of marine aggregates. Limnology and Oceanography, 65(6), 1264–1285.

[ece37119-bib-0044] Mayor, D. J. , Sanders, R. , Giering, S. L. C. , & Anderson, T. R. (2014). Microbial gardening in the ocean's twilight zone. BioEssays : News and Reviews in Molecular, Cellular and Developmental Biology, 36, 1132–1137. 10.1002/bies.201400100 PMC4278546 25220362

[ece37119-bib-0045] Mayzaud, P. , Boutoute, M. , Gasparini, S. , & Mousseau, L. (2014). Lipids and fatty acid composition of particulate matter in the North Atlantic: Importance of spatial heterogeneity, season and community structure. Marine Biology, 161, 1951–1971. 10.1007/s00227-014-2476-9

[ece37119-bib-0046] Moi, I. M. , Leow, A. T. C. , Ali, M. S. M. , Rahman, R. N. Z. R. A. , Salleh, A. B. , & Sabri, S. (2018). Polyunsaturated fatty acids in marine bacteria and strategies to enhance their production. Applied Microbiology and Biotechnology, 102, 5811–5826. 29749565 10.1007/s00253-018-9063-9

[ece37119-bib-0047] Neal, A. C. , Prahl, F. G. , Eglinton, G. , O'Hara, S. C. M. , & Corner, E. D. S. (1986). Lipid changes during a planktonic feeding sequence involving unicellular algae, *Elminius* nauplii and adult *Calanus* . Journal of the Marine Biological Association of the United Kingdom, 66, 1–13.

[ece37119-bib-0048] Okuyama, H. , Orikasa, Y. , & Nishida, T. (2008). Significance of antioxidative functions of eicosapentaenoic and docosahexaenoic acids in marine microorganisms. Applied and Environmental Microbiology, 74, 570–574. 18065628 10.1128/AEM.02256-07 PMC2227742

[ece37119-bib-0049] Parekh, P. , Follows, M. J. , & Boyle, E. A. (2005). Decoupling of iron and phosphate in the global ocean. Global Biogeochemical Cycles, 19(2). 10.1029/2004GB002280

[ece37119-bib-0050] Ploug, H. , & Bergkvist, J. (2015). Oxygen diffusion limitation and ammonium production within sinking diatom aggregates under hypoxic and anoxic conditions. Marine Chemistry, 176, 142–149. 10.1016/j.marchem.2015.08.012

[ece37119-bib-0051] Ploug, H. , Iversen, M. H. , Koski, M. , & Buithenhuis, E. T. (2008). Production, oxygen respiration rates, and sinking velocity of copepods fecal pellets: Direct measurments of ballasting by opal and calcite. Limnology and Oceanography, 53, 469–476.

[ece37119-bib-0052] Pond, D. W. , Priddle, J. , Sargent, J. R. , & Watkins, J. L. (1995). Laboratory studies of assimilation and egestion of algal lipid by Antarctic krill — Methods and initial results. Journal of Experimental Marine Biology and Ecology, 187, 253–268.

[ece37119-bib-0053] Poulsen, L. , & Iversen, M. (2008). Degradation of copepod fecal pellets: Key role of protozooplankton. Marine Ecology Progress Series, 367, 1–13.

[ece37119-bib-0054] Proud, R. , Cox, M. J. , & Brierley, A. S. (2017). Biogeography of the global ocean's mesopelagic zone. Current Biology, 27, 113–119. 10.1016/j.cub.2016.11.003 28017608

[ece37119-bib-0055] Romero‐Romero, S. , Ka'apu‐Lyons, C. A. , Umhau, B. P. , Benitez‐Nelson, C. R. , Hannides, C. C. S. , Close, H. G. , & Drazen, J. C. , et al. (2020). Deep zooplankton rely on small particles when particle fluxes are low. Limnology and Oceanography Letters, 5(6), 410–416. 10.1002/lol2.10163

[ece37119-bib-0056] Russell, N. J. , & Nichols, D. S. (1999). Polyunsaturated fatty acids in marine bacteria – A dogma rewritten. Microbiology (Reading, England), 145(Pt 4), 767–779. 10.1099/13500872-145-4-767 10220156

[ece37119-bib-0057] Schmidt, K. , Atkinson, A. , Steigenberger, S. , Fielding, S. , Lindsayc, M. C. M. , Pond, D. W. , Tarling, G. A. , Klevjer, T. A. , Allen, C. S. , Nicol, S. , & Achterberg, E. P. (2011). Seabed foraging by Antarctic krill: Implications for stock assessment, bentho‐pelagic coupling, and the vertical transfer of iron. Limnology and Oceanography, 56(4), 1411–1428. 10.4319/lo.2011.56.4.1411

[ece37119-bib-0058] Schmitz, O. J. , Raymond, P. A. , Estes, J. A. , Werner, A. , Holtgrieve, G. W. , Ritchie, M. E. , Schindler, D. E. , et al. (2013). Animating the carbon cycle. Ecosystems, 17, 344–359.

[ece37119-bib-0059] Shulse, C. N. , & Allen, E. E. (2010). Diversity and distribution of microbial long‐chain fatty acid biosynthetic genes in the marine environment. Environmental Microbiology, 13(3), 685–695. 10.1111/j.1462-2920.2010.02373.x 21105981

[ece37119-bib-0060] Shulse, C. N. , & Allen, E. E. (2011). Widespread occurrence of secondary lipid biosynthesis potential in microbial lineages. PLoS One, 6, e20146 10.1371/journal.pone.0020146 21629834 PMC3098273

[ece37119-bib-0061] Siegel, D. A. (1998). Resource competition in a discrete environment: Why are plankton distributions paradoxical? Limnology and Oceanography, 43, 1133–1146.

[ece37119-bib-0062] Stoecker, D. K. , & Capuzzo, J. M. (1990). Predation on protozoa: Its importance to zooplankton. Journal of Plankton Research, 12, 891–908. 10.1093/plankt/12.5.891

[ece37119-bib-0063] Suzuki, H. , Sasaki, H. , & Fukuchi, M. (2003). Loss processes of sinking fecal pellets of zooplankton in the mesopelagic layers of the Antarctic Marginal Ice zone. Journal of Oceanography, 59, 809–818. 10.1023/B:JOCE.0000009572.08048.0d

[ece37119-bib-0064] Tanoue, E. , Handa, N. , & Sakugawa, H. (1982). Difference of the chemical composition of organic matter between fecal pellet of *Euphausia superba* and its feed, *Dunaliella tertiolecta* . Transaction of the Tokyo University of Fisheries, 5, 189–196.

[ece37119-bib-0065] Tanoue, E. , & Hara, S. (1986). Ecological implications of fecal pellets produced by the Antartic krill *Euphausia superba* in the Antarctic Ocean. Marine Biology, 91, 359–369.

[ece37119-bib-0066] Tarling, G. A. , & Fielding, S. (2016). Swarming and behaviour in Antarctic krill In Siegel V. (Ed.), Biology and ecology of Antarctic krill (pp. 279–319). Springer.

[ece37119-bib-0067] Thor, P. , & Dam, H. G. (2003). Fate of organic carbon released from decomposing copepod fecal pellets in relation to bacterial production and ectoenzymatic activity. Aquatic Microbial Ecology, 33, 279–288.

[ece37119-bib-0068] Troch, M. D. , Steinarsdóttir, M. B. , Chepurnov, V. A. , & Olafsson, E. (2005). Grazing on diatoms by harpacticoid copepods: Species‐specific density‐dependent uptake and microbial gardening. Aquatic Microbial Ecology, 39, 135–144.

[ece37119-bib-0069] Turner, J. T. (2015). Zooplankton fecal pellets, marine snow, phytodetritus and the ocean's biological pump. Progress in Oceanography, 130, 205–248. 10.1016/j.pocean.2014.08.005

[ece37119-bib-0070] Vanderploeg, H. A. (1994). Zooplankton particle selection and feeding mechanisms. The biology of particles in aquatic systems (pp. 205–234). Lewis.

[ece37119-bib-0071] Volk, T. , & Hoffert, M. (1985). Ocean carbon pumps: Analysis of relative strengths and efficiencies in ocean‐driven atmospheric CO_2_ changes Sandquist E. & Broecker W. (Eds.), In The carbon cycle and atmospheric CO_2_: natural variations Archean to present (pp. 99–110). American Geophysical Union.

[ece37119-bib-0072] Wakeham, S. G. , Hedges, J. I. , Lee, C. , Peterson, M. L. , & Hernes, P. J. (1997). Compositions and transport of lipid biomarkers through the water column and surficial sediments of the equatorial Pacific Ocean. Deep‐Sea Research Part II: Topical Studies in Oceanography, 44, 2131–2162.

[ece37119-bib-0073] Wassmann, P. , Hansen, L. , Andreassen, I. J. , Riser, C. W. , Urban‐Rich, J. , & Båmstedt, U. (1999). Distribution and sedimentation of faecal on the Nordvestbanken shelf, northern Norway, in 1994. Sarsia, 84, 239–253. 10.1080/00364827.1999.10420429

[ece37119-bib-0074] Werner, I. (2000). Faecal pellet production by Arctic under‐ice amphipods – Transfer of organic matter through the ice/water interface. Hydrobiologia, 426, 89–96. 10.1023/A:1003984327103

[ece37119-bib-0075] Wilson, S. , Steinberg, D. , & Buesseler, K. (2008). Changes in fecal pellet characteristics with depth as indicators of zooplankton repackaging of particles in the mesopelagic zone of the subtropical and subarctic North Pacific Ocean. Deep‐Sea Research Part Ii‐Topical Studies in Oceanography, 55, 1636–1647.

[ece37119-bib-0076] Yoshida, K. , Hashimoto, M. , Hori, R. , Adachi, T. , Okuyama, H. , Orikasa, Y. , et al. (2016). Bacterial long‐chain polyunsaturated fatty acids: Their biosynthetic genes, functions, and practical use. Marine Drugs, 14, 94 10.3390/md14050094 PMC4882568 27187420

[ece37119-bib-0077] Zimmerman, A. R. , & Canuel, E. A. (2001). Bulk organic matter and lipid biomarker composition of Chesapeake Bay surficial sediments as indicators of environmental processes. Estuarine, Coastal and Shelf Science, 53, 319–341.

