# Supplementary material for: Implications for the mesopelagic microbial gardening hypothesis as determined by experimental fragmentation of Antarctic krill fecal pellets
Source: Ecol Evol. 2020 Dec 28;11(2):1023–36. doi: 10.1002/ece3.7119 (PMC7820144; doi:10.1002/ece3.7119)
Supplement: Supplementary file 1 — Supplementary Material [file ECE3-11-1023-s001.docx]

**Supporting Information**

**Implications for the mesopelagic microbial gardening hypothesis as determined by experimental fragmentation of Antarctic krill faecal pellets**

This file contains three extra and four tables graphics to support the main text:

**Fig. S1**. Photo of microrespiration vials containing pellets prior to experiment beginning

**Fig. S2**. Oxygen concentration over time with linear regressions

**Table S1**. Results of linear regression fits between oxygen concentration and time.

**Table S2**. Results of linear regressions and oxygen uptake rates when data removed if O_2_ concentration went < 100 µM.

**Table S3.** Effect of removing data on mean POC turnover rate where the coefficient p-values were > 0.05 for O_2_ uptake rates.

**Table S4.** Results of POC turnover rate t-tests with and without exclusions of vials with non-significant O_2_ uptake rates.

**Fig. S3**. Histogram of pellet length with and without fragmentation.

**
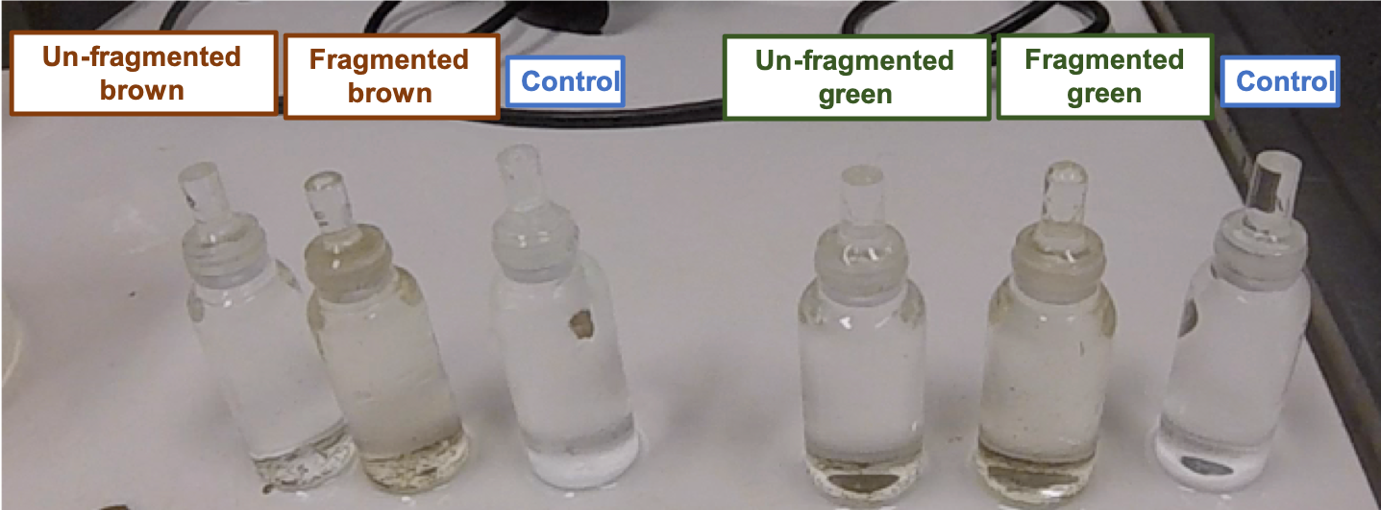
Supporting figures**

**Fig. S1.** Micro-respiration vials post-fragmentation prior to being placed in the water bath and the experiment beginning. Pellets can be seen in the base of the experimental vials. The water is coloured in the fragmented vials showing the release of DOM from the faecal pellets upon fragmentation.

**Figure S2.** Oxygen concentration over time in each via from the 4 replicate experiments (panels 1-4). Solid lines and points are unfragmented, large pellets, dashed lines and triangles are small, fragmented pellets and the colours represent the green or brown groupings of pellets. Blue lines and points are the control vials without pellets. The lines are linear regressions and the slopes of these regressions are the oxygen uptake rates in Fig. 2c in the main text. Note these data are not yet normalised to POC content in each vial.

| **Experiment** | **Vial code** | **FP length** | **FP colour** | **O_2_ uptake (µmol L^-1^ h^-1^)** | **p-value intercept** | **p-value slope** | **R^2^** |
| --- | --- | --- | --- | --- | --- | --- | --- |
| 1 | C1 | - | - | 0.558 | 0.000 | 0.042 | 0.917 |
| 1 | C2 | - | - | 0.658 | 0.001 | 0.080 | 0.847 |
| 1 | BL | Long | Brown | 1.842 | 0.009 | 0.143 ^a,b^ | 0.735 |
| 1 | GL | Long | Green | 1.375 | 0.002 | 0.057^a^ | 0.889 |
| 1 | BS | Short | Brown | 2.425 | 0.005 | 0.049 | 0.904 |
| 1 | GS | Short | Green | 2.288 | 0.016 | 0.138 ^a,b^ | 0.743 |
| 2 | C1 | - | - | 0.712 | 0.000 | 0.028 | 0.944 |
| 2 | C2 | - | - | 0.595 | 0.000 | 0.004 | 0.992 |
| 2 | BL | Long | Brown | 3.240 | 0.002 | 0.012 | 0.977 |
| 2 | GL | Long | Green | 3.191 | 0.000 | 0.002 | 0.996 |
| 2* | BS | Short | Brown | 4.016 | 0.000 | 0.001 | 0.999 |
| 2* | GS | Short | Green | 3.567 | 0.003 | 0.014 | 0.972 |
| 3 | C1 | - | - | 0.248 | 0.000 | 0.189 | 0.658 |
| 3 | C2 | - | - | 0.363 | 0.000 | 0.011 | 0.978 |
| 3 | BL | Long | Brown | 3.143 | 0.014 | 0.082 ^a^ | 0.842 |
| 3 | GL | Long | Green | 2.658 | 0.002 | 0.023 | 0.955 |
| 3* | BS | Short | Brown | 4.312 | 0.016 | 0.052 | 0.899 |
| 3 | GS | Short | Green | 2.041 | 0.002 | 0.029 | 0.944 |
| 4 | C1 | - | - | 0.560 | 0.001 | 0.171 | 0.687 |
| 4 | C2 | - | - | 0.615 | 0.001 | 0.170 | 0.688 |
| 4 | BL | Long | Brown | 1.856 | 0.000 | 0.008 | 0.984 |
| 4 | GL | Long | Green | 3.000 | 0.001 | 0.009 | 0.982 |
| 4* | BS | Short | Brown | 3.400 | 0.000 | 0.001 | 0.998 |
| 4 | GS | Short | Green | 3.188 | 0.000 | 0.003 | 0.995 |

**Table S1.** Results of the linear regression fits of oxygen concentration with time. C1 and C2 refer to the control vials with no pellets. The slope of the regression is shown as O_2_ uptake (µmol L^-1^ h^-1^). The p-values for the intercept and coefficient (here time in hours) are also given and the Adjusted R^2^. The regressions were computed in R. Note, all intercept p-values were below the typical significant value of 0.05.

^a^Four of the treatment vials (i.e. not controls) had coefficient (time) p-values for the linear regression of > 0.05 to 2.d.p.

^b^Two of these p-values were > 0.1.

*Vials where O_2_ concentrations reached < 100 µmol by the final reading (see Table S2).

| **Experiment** | **Vial code** | **O_2_ uptake (µmol L^-1^ h^-1^)** | **p-value intercept** | **p-value slope** | **R^2^** | **POC turnover (d^-1^)** |
| --- | --- | --- | --- | --- | --- | --- |
| 2 | BS | 4.167 | 0.001 | 0.019 | 0.999 | 0.040 |
| 2 | GS | 4.271 | 0.019 | 0.048 | 0.994 | 0.059 |
| 3 | BS | 4.879 | 0.115 | 0.266 | 0.835 | 0.041 |
| 4 | BS | 3.593 | 0.000 | 0.010 | 0.999 | 0.042 |

**Table S2.** Results of the linear regression fits of oxygen concentration with time for vials with O_2_ concentrations reached < 100 µmol when the data < 100 O_2_ µmol are removed (* in Table S1). POC turnover is also shown for comparison with Table 1 in the man manuscript.

**Table S3.** Mean POC turnover rates (d^-1^ ± standard deviation) when all data are used as reported in the main manuscript (n=16), when data for vials with linear regressions for which the coefficient p-value was > 0.1 are removed (n=14, i.e. all data remaining had coefficient p-values <0.1), and when data for vials with coefficient p-value > 0.05 are removed (n=12, i.e. all data remaining had coefficient p-values <0.05).

| **Treatment** | **All data** | **p < 0.1** | **p < 0.05** |
| --- | --- | --- | --- |
| BL | 0.020 (± 0.004) | 0.019 (± 0.003) | 0.019 (± 0.004) |
| BS | 0.037 (± 0.002) | 0.037 (± 0.002) | 0.037 (± 0.002) |
| GL | 0.036 (± 0.013) | 0.036 (± 0.013) | 0.038 (± 0.015) |
| GS | 0.035 (± 0.018) | 0.030 (± 0.019) | 0.030 (± 0.019) |

**Table S4 a - c.** Results of Welch’s t-tests between POC turnover rates (given as means d^-1^) for different treatments. See Table S2 for standard deviations about the mean.

**a)** Using all data (n = 16)

| **Treatment 1** | **Treatment 2** | **Mean Treatment 1** | **Mean Treatment 2** | **t-test p-value** |
| --- | --- | --- | --- | --- |
| BL | BS | 0.020 | 0.037 | 0.00 |
| BL | GL | 0.020 | 0.036 | 0.09 |
| BL | GS | 0.020 | 0.035 | 0.21 |
| BS | GL | 0.037 | 0.036 | 0.90 |
| BS | GS | 0.037 | 0.035 | 0.86 |
| GL | GS | 0.036 | 0.035 | 0.94 |

**b)** Removing slopes where p > 0.1 (n = 14)

| **Treatment 1** | **Treatment 2** | **Mean Treatment 1** | **Mean Treatment 2** | **t-test p-value** |
| --- | --- | --- | --- | --- |
| BL | BS | 0.019 | 0.037 | 0.00 |
| BL | GL | 0.019 | 0.036 | 0.07 |
| BL | GS | 0.019 | 0.030 | 0.41 |
| BS | GL | 0.037 | 0.036 | 0.90 |
| BS | GS | 0.037 | 0.030 | 0.59 |
| GL | GS | 0.036 | 0.030 | 0.66 |

**c)** Removing slopes where p > 0.05 (n = 12)

| **Treatment 1** | **Treatment 2** | **Mean Treatment 1** | **Mean Treatment 2** | **t-test p-value** |
| --- | --- | --- | --- | --- |
| BL | BS | 0.019 | 0.037 | 0.07 |
| BL | GL | 0.019 | 0.038 | 0.14 |
| BL | GS | 0.019 | 0.030 | 0.42 |
| BS | GL | 0.037 | 0.038 | 0.90 |
| BS | GS | 0.037 | 0.030 | 0.59 |
| GL | GS | 0.038 | 0.030 | 0.58 |

**Figure S3.** Histograms of faecal pellet perimeter size (mm) of the four different faecal pellet fractions; long brown, short brown (fragmented), long green and short green (fragmented).
